# Determinants and survival benefits of achieving textbook outcome for intrahepatic cholangiocarcinoma in the era of neoadjuvant therapy

**DOI:** 10.3389/fonc.2026.1737204

**Published:** 2026-01-22

**Authors:** Jiawei Hu, Yihang Wang, Haoran Diao, Shuangda Miao, Xiaoxiao Zhang, Qi Li, Yanzhi Pan, Yun Jin, Yuanquan Yu, Jiangtao Li

**Affiliations:** Department of Hepatopancreatobiliary Surgery, The Second Affiliated Hospital, College of Medicine, Zhejiang University, Hangzhou, Zhejiang, China

**Keywords:** immunotherapy, intrahepatic cholangiocarcinoma, neoadjuvant therapy, prognostic model, targeted therapy, textbook outcomes

## Abstract

**Background:**

Intrahepatic cholangiocarcinoma (ICC) is a highly aggressive malignancy with a poor prognosis. Radical resection is the modality to cure patients with ICC. Thus, surgical quality is the key prognostic factor for survival. Textbook outcome (TO) is a multidimensional composite indicator reflecting surgical care quality. However, the association between neoadjuvant therapies—particularly those incorporating targeted and/or immunotherapeutic agents into chemotherapy regimens—and the attainment of TO in ICC remains unclear and warrants further investigation.

**Materials and methods:**

This retrospective study analyzed 187 patients with ICC who underwent curative resection. TO was defined as the simultaneous achievement of R0 resection, with no perioperative blood transfusion, no postoperative complications, no mortality within 30 days, no unplanned readmission within 30 days, and a postoperative length of stay not exceeding the 75th percentile. Logistic regression was used to identify factors associated with TO, with further analysis focused on the role of neoadjuvant therapy. Cox regression was used to evaluate prognostic factors for overall survival (OS), and a prognostic nomogram incorporating TO was developed and validated.

**Results:**

TO was achieved in 53 patients (28.3%), which was significantly associated with improved OS (*p* = 0.003) and recurrence-free survival (*p* < 0.001). Multivariable analysis identified neoadjuvant therapy [odds ratio (OR) = 2.687, *p* = 0.014], higher body mass index, higher albumin levels, lower carcinoembryonic antigen levels, and reduced blood loss as independent predictors of TO. Combination neoadjuvant regimens (chemotherapy plus targeted/immunotherapy; OR = 2.647, *p* = 0.009) were the primary contributors to this positive association. A nomogram integrating TO, lymph node metastasis, prothrombin time, and adjuvant therapy demonstrated excellent predictive accuracy for survival (1-year area under the curve = 0.891).

**Conclusion:**

Achieving TO is associated with significantly improved survival in patients with ICC. Combined neoadjuvant therapy, including targeted or immunotherapy, is an independent positive predictor of TO, which challenges conventional perspectives. The proposed TO-integrated nomogram is a practical tool for prognostic prediction and surgical quality assessment.

## Introduction

Intrahepatic cholangiocarcinoma (ICC) is a malignant tumor that originates from the intrahepatic biliary epithelium, accounting for approximately 10%–20% of all cholangiocarcinoma cases. It is the second most common primary liver malignancy, constituting approximately 10% of all primary liver cancers (1, 2). The global incidence of ICC has been significantly increasing in recent years (3). The disease is highly aggressive and associated with a poor prognosis, with postoperative 3- and 5-year overall survival (OS) rates of only 30% and 18%, respectively (4). Complete surgical resection remains the primary curative treatment for ICC (5); however, only approximately 30% of patients are considered eligible for resection at initial diagnosis (6). Recent advances in neoadjuvant therapy, including chemotherapy, targeted therapy, and immunotherapy, have provided opportunities for conversion surgery in initially unresectable or borderline resectable cases, which significantly expand the pool of surgical candidates (7–10).

Due to its anatomical location, ICC resection frequently involves complex vascular and biliary reconstruction, thereby presenting considerable technical challenges and perioperative risks (11, 12). Surgical management has become more complicated due to the expanding use of neoadjuvant therapies. The dual aspects of antitumor efficacy and potential drug-induced liver injury contribute substantially to unclear surgical outcomes, underscoring the critical importance of precise surgical quality assessment in this context (13, 14). Several studies have indicated that surgical quality directly affects patient prognosis. For example, R0 resection has significantly improved survival rates (15, 16). However, current surgical outcome assessments often rely on isolated indicators (17), which lack integration and fail to provide a comprehensive reflection of overall treatment quality, despite their clinical relevance.

Textbook outcome (TO) is a multidimensional composite endpoint that incorporates multiple indicators such as R0 resection, no perioperative blood transfusion, no complications, no prolonged postoperative hospital stay, no unplanned readmission within 30 days, and no 30-day mortality (18, 19). TO was initially applied in colorectal cancer and is now increasingly adopted in ICC surgical evaluation and strongly correlated with improved long-term survival (18–21). Traditionally, chemotherapy-based neoadjuvant therapy was considered a negative predictor for TO, with studies indicating its potential to increase surgical risk (19, 22). However, with rapid advancements in targeted and immunotherapy, this perception warrants reevaluation. These novel therapies, characterized by higher response rates and improved safety profiles, may improve resectability, reduce surgical difficulty, and consequently facilitate TO achievement (23).

Therefore, using a cohort of patients with ICC and detailed neoadjuvant therapy information, this study aimed to investigate the association between TO and patient prognosis, identify clinical factors that are independently associated with TO achievement, and specifically assess the impact of neoadjuvant therapy—particularly regimens that incorporate targeted and immunotherapeutic agents—on TO. Furthermore, we developed and validated a TO-integrated prognostic prediction model to provide a practical tool to support clinical decision-making and surgical quality assessment.

## Materials and methods

This retrospective cohort study consecutively enrolled patients who underwent resection for ICC at the Second Affiliated Hospital of Zhejiang University School of Medicine from May 2011 to September 2024. Inclusion criteria were pathological confirmation of ICC. Exclusion criteria were concomitant other malignancies, incomplete follow-up data, and missing information on tumor characteristics or surgical procedures.

Data were systematically collected on patient demographics [age, sex, and body mass index (BMI]), underlying disease (e.g., diabetes and hypertension), liver function parameters, and American Society of Anesthesiologists classification, tumor characteristics (e.g., size, T stage, differentiation, vascular invasion, lymph node metastasis, and distant metastasis), laboratory values [e.g., serum albumin (ALB), total bilirubin, prothrombin time (PT), carcinoembryonic antigen (CEA), carbohydrate antigen 19-9 (CA19-9), and alpha-fetoprotein (AFP)], and surgical details (e.g., scope of surgery, surgical method, lymph node dissection, operation time, and intraoperative blood loss). Further, information on neoadjuvant and adjuvant therapies was recorded, along with surgical quality indicators and postoperative survival outcomes.

TO was defined as the simultaneous achievement of the following six criteria: R0 resection, no perioperative transfusion, no postoperative complications, no 30-day mortality, no unplanned readmission within 30 days, and postoperative length of stay not exceeding the 75th percentile of the cohort (18, 19). Neoadjuvant therapy refers to preoperative systemic treatment, including chemotherapy, chemotherapy with targeted therapy, or immunotherapy, for patients with borderline resectable tumors. It may be considered for those with a large tumor burden, multifocal disease, or major vascular involvement. Additionally, for patients with technically resectable intrahepatic tumors but high-risk features for recurrence, neoadjuvant therapy may also be considered. The decision to administer neoadjuvant therapy or proceed directly with surgery also depends on the patient’s overall health, individual preferences, and other factors (24). Resections were classified as major (≥3 Couinaud’s segments) or minor (<3 segments) (25). Intraoperative blood loss and operation time were dichotomized according to median values.

The Shapiro–Wilk test was used to test continuous variables for normality. Normally distributed variables were expressed as mean ± standard deviation and compared using analysis of variance; non-normal variables were reported as median (interquartile range) and compared using non-parametric tests. Categorical variables were expressed as frequencies and percentages, compared with χ^2^ or Fisher’s exact test. Baseline characteristics and perioperative outcomes were compared between the TO and non-TO groups. Reasons for not achieving TO were analyzed. Univariate and multivariate logistic regression analyses, with additional in-depth evaluation focusing on neoadjuvant therapy, were conducted to identify factors associated with TO. Based on TO status, univariate and multivariate Cox regression analyses developed a prognostic model, visualized via a nomogram. Receiver operating characteristic (ROC) curves were used to assess discriminative ability. R (version 4.3.1) was used for analyses, with *p*-values of <0.05 indicating significance.

## Results

### Baseline characteristics and textbook outcomes

The study included 187 patients with a mean age of 61.88 ± 10.06 years, of whom 107 (57.2%) were male and 80 (42.8%) were female. Among these patients, 56 (29.9%) received neoadjuvant therapy. Of those, 13 patients (23.2%) received chemotherapy alone, while 43 patients (76.8%) received chemotherapy combined with targeted therapy or immunotherapy. A TO was achieved in 53 patients, constituting 28.3% of the entire cohort. Compared with patients who did not achieve TO, those who achieved TO had better nutritional reserves, as reflected by higher BMI (*p* = 0.008) and ALB (*p* = 0.001), as well as lower malignant potential indicated by CEA (*p* = 0.003) and reduced lymph node metastasis (*p* = 0.012). The scope of surgery exhibited no significant difference (*p* = 1.000); however, the TO group demonstrated a higher proportion of laparoscopic surgeries (*p* = 0.002), along with reduced intraoperative blood loss (*p* < 0.001) and shorter operation time (*p* = 0.003). Furthermore, the TO group received neoadjuvant therapy at a significantly higher rate (*p* = 0.007) (**Table 1**).

**Table 1:** Clinical characteristics of the total, textbook outcome (TO), and non-textbook outcome (non-TO) cohorts.

Characteristics	Total population N = 187	Non-TO N = 134	TO N = 53	*P*-value
Age, years, mean (SD)	61.88 (10.06)	61.67 (9.75)	62.40 (10.89)	0.658
Sex, n (%)				0.700
Female	80 (42.8)	59 (44.0)	21 (39.6)	
Male	107 (57.2)	75 (56.0)	32 (60.4)	
BMI, kg/m^2^, mean (SD)	22.88 (3.07)	22.51 (3.05)	23.82 (2.95)	0.008
Underlying disease, n (%)				0.535
No	128 (68.4)	94 (70.1)	34 (64.2)	
Yes	59 (31.6)	40 (29.9)	19 (35.8)	
Child–Pugh score, n (%)				0.191
A	170 (90.9)	119 (88.8)	51 (96.2)	
B	17 (9.1)	15 (11.2)	2 (3.8)	
Total bilirubin, n (%)				0.127
<20 μmol/L	151 (80.7)	104 (77.6)	47 (88.7)	
≥20 μmol/L	36 (19.3)	30 (22.4)	6 (11.3)	
Albumin, n (%)				0.001
≤35 g/L	34 (18.2)	33 (24.6)	1 (1.9)	
>35 g/L	153 (81.8)	101 (75.4)	52 (98.1)	
Prothrombin time, n (%)				0.379
≤13 s	91 (48.7)	62 (46.3)	29 (54.7)	
>13 s	96 (51.3)	72 (53.7)	24 (45.3)	
Carcinoembryonic antigen, n (%)				0.003
<5 ng/mL	139 (74.3)	91 (67.9)	48 (90.6)	
≥5 ng/mL	48 (25.7)	43 (32.1)	5 (9.4)	
Carbohydrate antigen 19-9, n (%)				0.283
<37 U/mL	75 (40.1)	50 (37.3)	25 (47.2)	
≥37 U/mL	112 (59.9)	84 (62.7)	28 (52.8)	
Alpha-fetoprotein, n (%)				0.205
<25 ng/mL	180 (96.3)	127 (94.8)	53 (100.0)	
≥25 ng/mL	7 (3.7)	7 (5.2)	0 (0.0)	
Tumor diameter, n (%)				0.894
<5 cm	102 (54.5)	74 (55.2)	28 (52.8)	
≥5 cm	85 (45.5)	60 (44.8)	25 (47.2)	
T stage, n (%)				0.268
T3–T4	32 (17.1)	26 (19.4)	6 (11.3)	
T1–T2	155 (82.9)	108 (80.6)	47 (88.7)	
Distant metastasis, n (%)				1.000
No	186 (99.5)	133 (99.3)	53 (100.0)	
Yes	1 (0.5)	1 (0.7)	0 (0.0)	
Lymph node metastasis, n (%)				0.012
No	124 (66.3)	81 (60.4)	43 (81.1)	
Yes	63 (33.7)	53 (39.6)	10 (18.9)	
AJCC stage, n (%)				0.104
I–II	97 (51.9)	64 (47.8)	33 (62.3)	
III–IV	90 (48.1)	70 (52.2)	20 (37.7)	
Degree of differentiation, n (%)				0.644
Low	92 (49.2)	64 (47.8)	28 (52.8)	
Mild–high	95 (50.8)	70 (52.2)	25 (47.2)	
Vascular invasion, n (%)				0.489
No	118 (63.1)	82 (61.2)	36 (67.9)	
Yes	69 (36.9)	52 (38.8)	17 (32.1)	
Surgical method, n (%)				0.002
Open	160 (85.6)	122 (91.0)	38 (71.7)	
Laparoscopic	27 (14.4)	12 (9.0)	15 (28.3)	
Lymph node dissection, n (%)				0.094
No	19 (10.2)	10 (7.5)	9 (17.0)	
Yes	168 (89.8)	124 (92.5)	44 (83.0)	
Scope of surgery, n (%)				1.000
Major	166 (88.8)	119 (88.8)	47 (88.7)	
Minor	21 (11.2)	15 (11.2)	6 (11.3)	
Intraoperative bleeding, n (%)				<0.001
≤200 mL	95 (50.8)	53 (39.6)	42 (79.2)	
>200 mL	92 (49.2)	81 (60.4)	11 (20.8)	
Operation time, n (%)				0.003
≤285 min	96 (51.3)	59 (44.0)	37 (69.8)	
>285 min	91 (48.7)	75 (56.0)	16 (30.2)	
ASA classification, n (%)				0.462
≤2	181 (96.8)	131 (97.8)	50 (94.3)	
>2	6 (3.2)	3 (2.2)	3 (5.7)	
Neoadjuvant therapy, n (%)				0.007
No	131 (70.1)	102 (76.1)	29 (54.7)	
Yes	56 (29.9)	32 (23.9)	24 (45.3)	
Adjuvant therapy, n (%)				0.772
No	36 (19.3)	27 (20.1)	9 (17.0)	
Yes	151 (80.7)	107 (79.9)	44 (83.0)	

TO, textbook outcome; ASA, American Society of Anesthesiologists; AJCC, American Joint Committee on Cancer.

**Figure 1:**
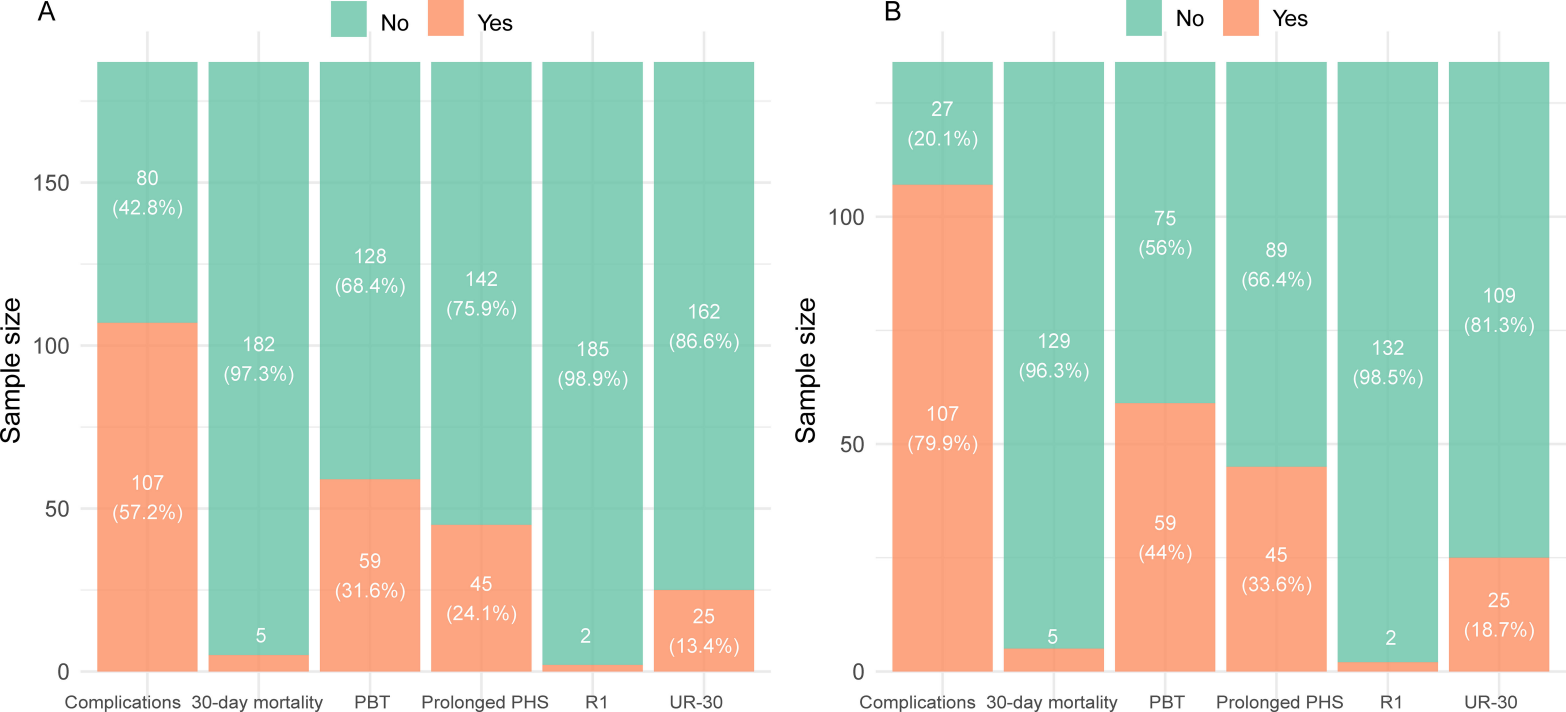
Textbook outcome components: presence of complications, 30-day mortality after surgery, perioperative blood transfusion (PBT), prolonged postoperative hospital stay (PHS), R1 resection margin (R1), and unplanned readmission within 30 days (UR-30) in the overall cohort **(A)** and among patients who failed to achieve textbook outcome **(B)**.

In the overall cohort, the primary reason for not achieving TO was complications (107 patients, 57.2%), followed by perioperative blood transfusion (59 patients, 31.6%), whereas 30-day mortality and positive resection margins exhibited a lesser impact (**Figure 1A**). Among patients who did not achieve TO, complications remained the predominant contributing factor (107 patients, 79.9%) (**Figure 1B**).

### Factors influencing TO

**Figure 2:**
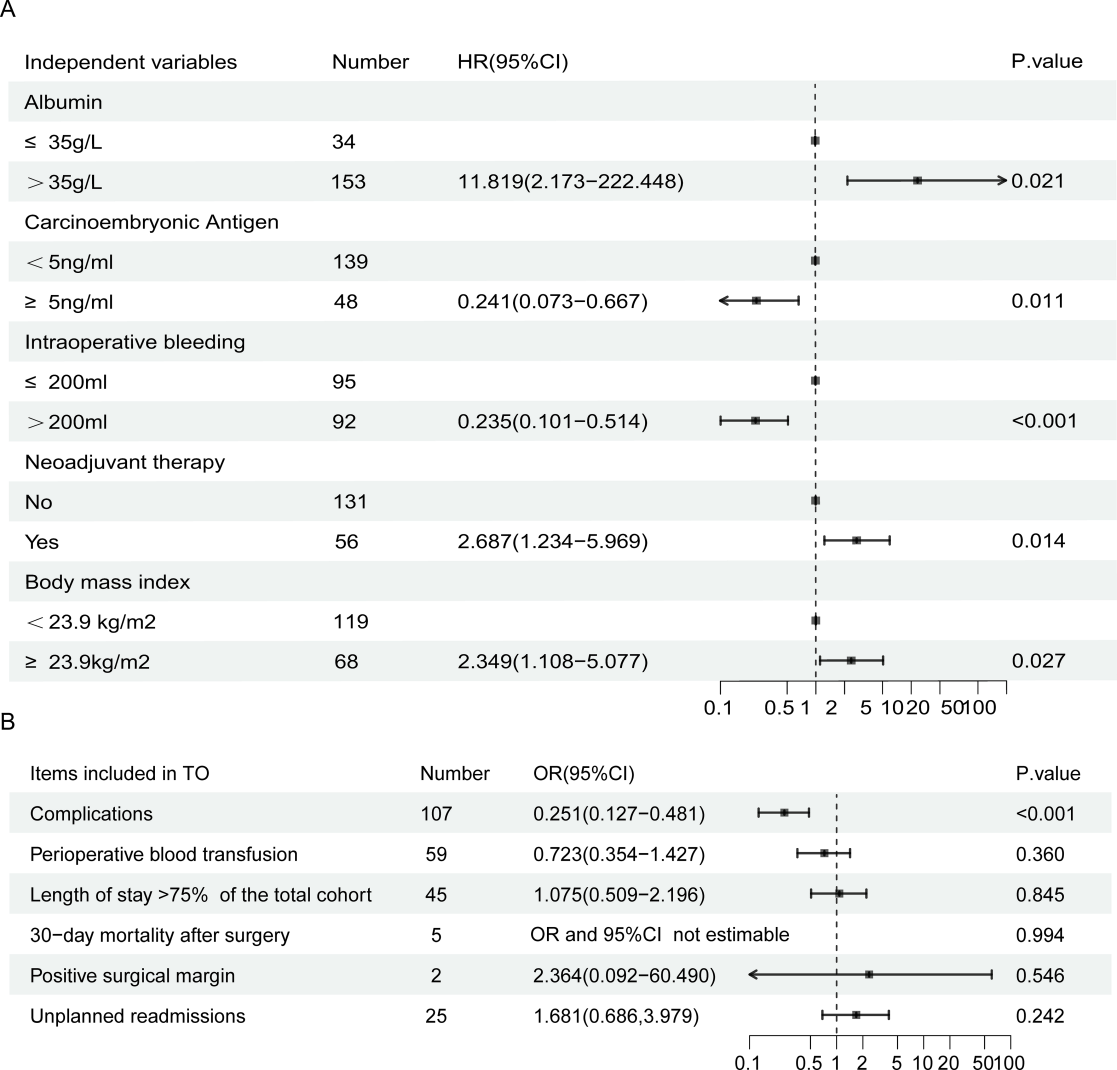
Forest plot of multivariable logistic regression analysis for textbook outcome **(A)** and univariable logistic regression analysis of neoadjuvant therapy on individual textbook outcome components **(B)**.

Univariate logistic regression analysis identified BMI, ALB, CEA, lymph node metastasis, surgical method, intraoperative bleeding, operation time, and neoadjuvant therapy as factors affecting the achievement of TO (all *p* < 0.05; **Table 2**). Multivariate analysis further revealed BMI [odds ratio (OR) = 2.349, 95% confidence interval (CI): 1.108–5.077, *p* = 0.027], ALB (OR = 11.819, 95% CI: 2.173–222.448, *p* = 0.021), CEA (OR = 0.241, 95% CI: 0.073–0.667, *p* = 0.011), intraoperative bleeding (OR = 0.235, 95% CI: 0.101–0.514, *p* < 0.001), and neoadjuvant therapy (OR = 2.687, 95% CI: 1.234–5.969, *p* = 0.014) as independent predictors of TO (**Table 2**). A forest plot illustrates these independent factors (**Figure 2A**).

**Table 2:** Logistic regression analysis of factors associated with textbook outcome.

Characteristics	Univariate analysis	Multivariate analysis		*P*-value
	*P*-value	OR	95% CI	
Age, years	0.237			
<65				
≥65				
Sex	0.583			
Female				
Male				
BMI	0.004			0.027
≤23.9 kg/m^2^		Reference	Reference	
>23.9 kg/m^2^		2.349	1.108−5.077	
Underlying disease	0.427			
No				
Yes				
Child–Pugh score	0.130			
A				
B				
Total bilirubin	0.090			
<20 μmol/L				
≥20 μmol/L				
Albumin	0.006			0.021
≤35 g/L		Reference	Reference	
>35 g/L		11.819	2.173–222.448	
Prothrombin time	0.298			
≤13 s				
>13 s				
Carcinoembryonic antigen	0.003			0.011
<5 ng/mL		Reference	Reference	
≥5 ng/mL		0.241	0.073–0.667	
Carbohydrate antigen 19-9	0.216			
<37 U/mL				
≥37 U/mL				
Alpha-fetoprotein	0.986			
<25 ng/mL				
≥25 ng/mL				
Tumor diameter	0.767			
<5 cm				
≥5 cm				
T stage	0.191			
T3–T4				
T1–T2				
Distant metastasis	0.988			
No				
Yes				
Lymph node metastasis	0.008			
No				
Yes				
AJCC stage	0.075			
I–II				
III–IV				
Degree of differentiation	0.532			
Low				
Mild–high				
Vascular invasion	0.391			
No				
Yes				
Surgical method	0.001			
Open				
Laparoscopic				
Lymph node dissection	0.058			
No				
Yes				
Scope of surgery	0.980			
Major				
Minor				
Intraoperative bleeding	<0.001			<0.001
≤200 mL		Reference	Reference	
>200 mL		0.235	0.101–0.514	
Operation time	0.002			
≤285 min				
>285 min				
ASA classification	0.248			
≤2				
>2				
Neoadjuvant therapy	0.005			0.014
No		Reference	Reference	
Yes		2.687	1.234–5.969	

CI, confidence interval; OR, odds ratio; ASA, American Society of Anesthesiologists; AJCC, American Joint Committee on Cancer.

Further analysis focused on the impact of neoadjuvant therapy on TO. Logistic regression was employed to examine its effects across six TO-related submetrics, with the results summarized in a forest plot (**Figure 2B**). Neoadjuvant therapy was significantly related to fewer postoperative complications (**Figure 2B**). Further composition analysis showed that, among patients who received neoadjuvant therapy, combination therapy was significantly associated with the achievement of TO (OR = 2.647, 95% CI: 1.277–5.482, *p* = 0.009), whereas chemotherapy alone was not significantly associated with TO achievement (*p* = 0.200).

### Survival and prognostic modeling

**Figure 3:**
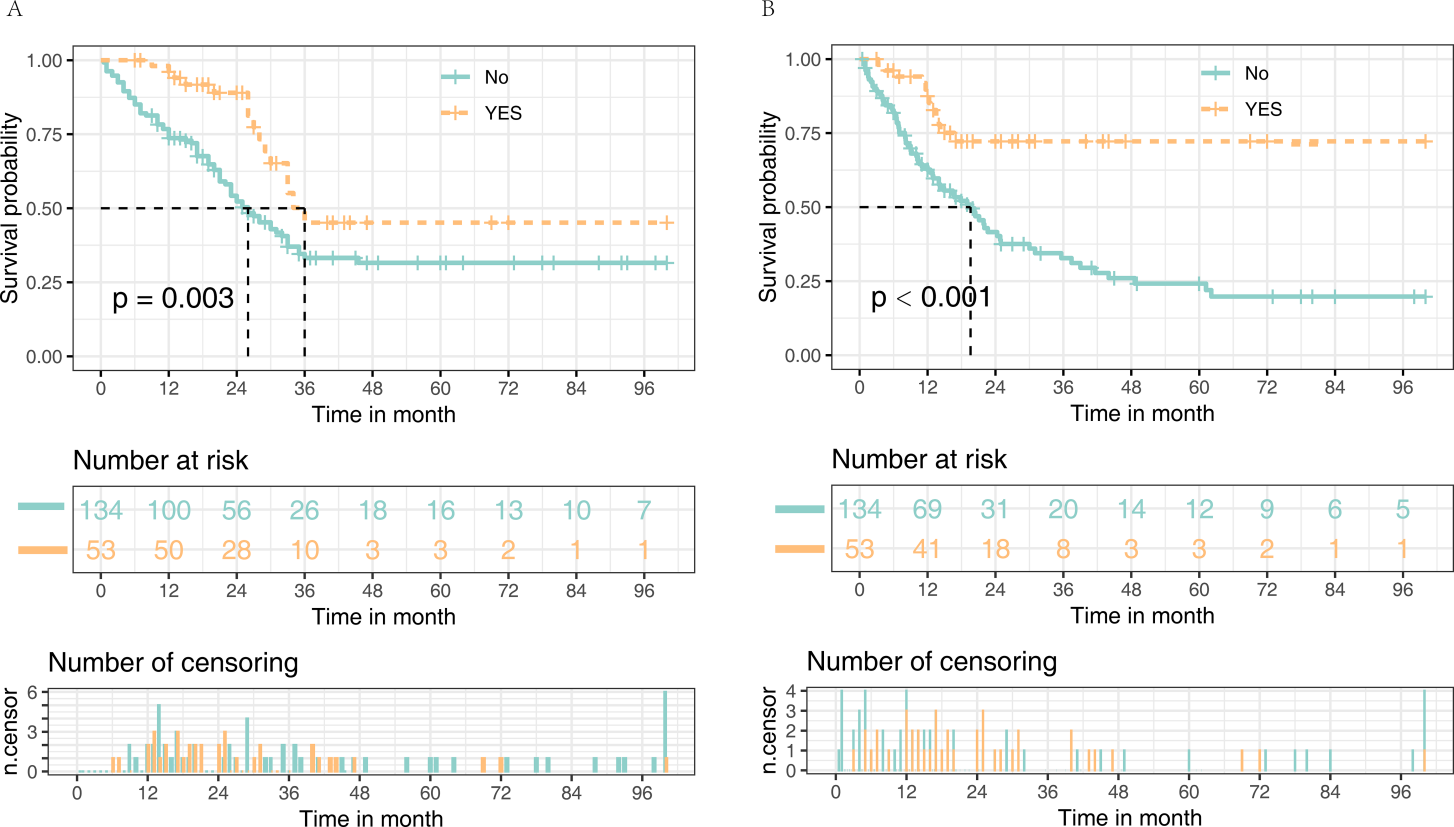
Kaplan–Meier curves comparing overall survival **(A)** and recurrence-free survival **(B)** in patients who achieved textbook outcome versus those who did not.

Median survival was 30 months, with a median follow-up of 37 months. The Kaplan–Meier analysis revealed that TO was associated with better OS (*p* = 0.003) and recurrence-free survival (RFS) (*p* < 0.001) (**Figure 3**). Univariate analysis identified PT, CEA, AFP, lymph node metastasis, American Joint Committee on Cancer stage, TO, operation time, and adjuvant therapy as prognostic factors (all *p* < 0.05). Multivariate Cox regression confirmed PT [hazard ratio (HR) = 1.746, 95% CI: 1.118–2.728, *p* = 0.014], lymph node metastasis (HR = 3.055, 95% CI: 1.981–4.712, *p* < 0.001), TO (HR = 0.559, 95% CI: 0.315–0.991, *p* = 0.047), and adjuvant therapy (HR = 0.301, 95% CI: 0.192–0.473, *p* < 0.001) as independent predictors (**Table 3**).

**Table 3:** Survival analysis based on textbook outcome status in patients with intrahepatic cholangiocarcinoma after surgery.

Characteristics	Univariate analysis	Multivariate analysis		*P*-value
	*P*-value	HR	95% CI	
Age, years	0.988			
<65				
≥65				
Sex	0.123			
Female				
Male				
BMI	0.422			
≤23.9 kg/m^2^				
>23.9 kg/m^2^				
Underlying disease	0.645			
No				
Yes				
Child–Pugh score	0.820			
A				
B				
Total bilirubin	0.927			
<20 μmol/L				
≥20 μmol/L				
Albumin	0.680			
≤35 g/L				
>35 g/L				
Prothrombin time	0.001			0.014
≤13 s		Reference	Reference	
>13 s		1.746	1.118–2.728	
Carcinoembryonic antigen	<0.001			
<5 ng/mL				
≥5 ng/mL				
Carbohydrate antigen 19-9	0.922			
<37 U/mL				
≥37 U/mL				
Alpha-fetoprotein	0.011			
<25 ng/mL				
≥25 ng/mL				
Tumor diameter	0.697			
<5 cm				
≥5 cm				
T stage	0.137			
T3–T4				
T1–T2				
Distant metastasis	0.404			
No				
Yes				
Lymph node metastasis	<0.001			<0.001
No		Reference	Reference	
Yes		3.055	1.981–4.712	
AJCC stage	0.005			
I–II				
III–IV				
Degree of differentiation	0.294			
Low				
Mild–high				
Vascular invasion	0.088			
No				
Yes				
Textbook outcome	0.004			0.047
No		Reference	Reference	
Yes		0.559	0.315–0.991	
Surgical method	0.376			
Open				
Laparoscopic				
Lymph node dissection	0.329			
No				
Yes				
Scope of surgery	0.180			
Major				
Minor				
Intraoperative bleeding	0.096			
≤200 mL				
>200 mL				
Operation time	0.024			
≤285 min				
>285 min				
ASA classification	0.644			
≤2				
>2				
Neoadjuvant therapy	0.143			
No				
Yes				
Adjuvant therapy	<0.001			<0.001
No		Reference	Reference	
Yes		0.301	0.192–0.473	

CI, confidence interval; HR, hazard ratio; ASA, American Society of Anesthesiologists; AJCC, American Joint Committee on Cancer.

**Figure 4:**
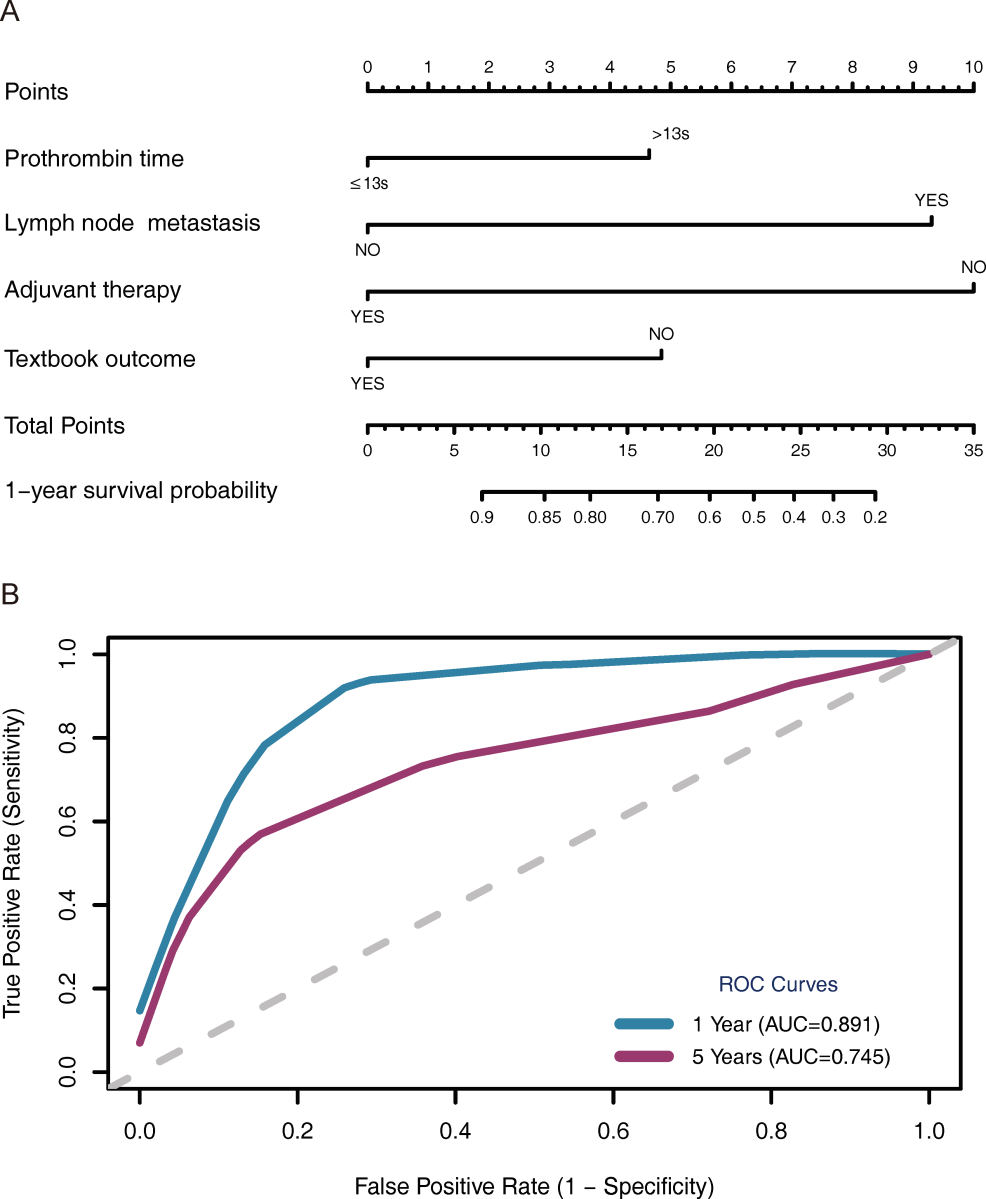
A nomogram for predicting postoperative overall survival in ICC patients based on textbook outcome **(A)** and its corresponding 1- and 5-year ROC curves **(B)**. ICC, intrahepatic cholangiocarcinoma; ROC, receiver operating characteristic.

Based on these independent predictors, a prognostic model that incorporates TO was developed and visualized as a nomogram for predicting survival for patients with ICC (**Figure 4A**). The model demonstrated good predictive ability, particularly for short-term prognosis, with area under the ROC curve values of 0.891 and 0.745 for 1- and 5-year survival, respectively (**Figure 4B**).

## Discussion

Comprehensive surgical quality assessment is crucial for patient outcomes due to the technical complexity of ICC resection and challenging postoperative recovery. TO, a composite endpoint that integrates multiple perioperative indicators, has gained broad acceptance in evaluating the quality of liver surgery (18, 19). Specific definitions vary, but numerous studies have consistently associated TO with significantly improved survival (19–21). This study further confirms that achieving TO markedly improves both OS and RFS in patients with ICC. Using multivariate logistic regression, we identified CEA level, intraoperative blood loss, ALB, BMI, and neoadjuvant therapy as independent factors affecting TO attainment. A prognostic nomogram that incorporates TO and other independent factors demonstrated favorable discrimination and calibration in internal validation.

A key finding of this study is that neoadjuvant therapy—particularly regimens that combine chemotherapy with targeted or immunotherapy—is a positive predictor for TO. Pawlik et al. analyzed patients with ICC who underwent surgery between 1993 and 2015 and revealed that neoadjuvant chemotherapy was associated with a lower TO rate (*p* = 0.04) (19). Separately, another study that incorporated data from 2000 to 2022 indicated that neoadjuvant therapy served as a negative factor for TO (22). This discrepancy may stem from evolving treatment paradigms. The majority of patients (77.19%) in our cohort received combination neoadjuvant therapy. Compared with chemotherapy alone, targeted and immunotherapies provide greater precision and an improved safety profile, which potentially facilitates tumor downstaging, reduces micrometastatic burden, and preserves liver function and overall condition, thereby enabling safer and more complete resection (26, 27). High-level clinical evidence, such as the KEYNOTE-966 and TOPAZ-1 phase III trials, which demonstrated survival benefits of immunotherapy–chemotherapy combinations in advanced biliary tract cancer, further support this perspective (28, 29). A phase II study by Guoming Shi et al. reported promising efficacy and safety for tislelizumab in combination with lenvatinib and GEMOX (gemcitabine and oxaliplatin) in the neoadjuvant setting (9). Further, neoadjuvant immunotherapy has been associated with higher TO rates in other malignancies, including esophageal cancer (23), which corroborates our findings. Notably, novel formulations (e.g., nab-paclitaxel and liposomal irinotecan) may improve both efficacy and safety by optimizing drug delivery (30, 31), potentially leading to better perioperative outcomes, even within the same class of chemotherapeutic agents.

Although neoadjuvant therapy significantly improved textbook outcomes for surgical patients in this study, some patients still experienced rapid disease progression shortly after treatment. In these cases, the tumors were often highly resistant to treatment and exhibited aggressive biological behavior. Preoperative disease progression can avoid unnecessary and ineffective surgery, thereby reducing the risk of early postoperative recurrence (32). This further emphasizes the clinical value of neoadjuvant therapy, as it aids in identifying patients who are unlikely to benefit from surgery and supports more informed decision-making.

Other factors associated with TO hold clinical relevance. CEA elevation is a biomarker of aggressive tumor biology and generally indicates an increased tumor burden, which may consequently elevate the complexity of surgical intervention (33, 34). ALB and BMI reflect nutritional and baseline status. Increased ALB levels have been associated with a higher rate of TO (35). Some studies have indicated that a BMI of ≥30 kg/m^2^ may negatively affect TO (36); however, in the present cohort, the mean BMI was 22.88 kg/m^2^, and no patient reached a BMI of 30 kg/m^2^. These findings indicate that population characteristics, regional variations, and other factors may influence the association between BMI and TO (37, 38), which warrants further investigation for clarity. Intraoperative blood loss is a critical determinant of TO, closely associated with perioperative transfusion, which is itself associated with increased infection risk and worse survival (39).

The prognostic model developed herein integrated TO, PT, lymph node metastasis, and adjuvant therapy, which demonstrates good predictive performance. The role of adjuvant therapy (e.g., capecitabine and S1) is well-established from phase III trials such as BILCAP and JCOG1202 (40, 41). Lymph node metastasis is a recognized poor prognostic factor, and adequate dissection (≥6 nodes) is crucial for accurate staging (42). Prolonged PT may indicate impaired liver function, tumor burden, and systemic inflammation (43).

Compared with previous studies, our work highlights the evolving landscape of neoadjuvant therapy, particularly the positive impact of combination targeted/immunotherapy on TO. The inclusion of variables spanning preoperative patient status, tumor features, surgical details, and perioperative treatments improves the model’s comprehensiveness and utility. Limitations include its single-center, retrospective design, limited sample size, and potential selection bias. Thus, validation through multicenter, larger cohorts is warranted. Heterogeneity in neoadjuvant regimens (e.g., drug combinations, cycles, and molecular subtypes) may affect outcomes (44, 45), requiring subgroup analyses with expanded samples. Furthermore, the lack of a globally standardized TO definition and the potential influence of institutional policies and recovery pathways on metrics, such as postoperative length of stay (46, 47), underscore the need for consensus-building.

## Conclusion

This study confirms that achieving TO significantly improves survival in patients with ICC and identifies CEA, intraoperative blood loss, ALB, BMI, and neoadjuvant therapy as independent influencing factors. Notably, this study is the first to report that neoadjuvant regimens that combine chemotherapy with targeted or immunotherapy improve TO attainment, which provides crucial evidence for neoadjuvant therapy strategies in ICC. The developed nomogram demonstrates robust prognostic predictive ability, thereby offering a practical tool for individualized clinical decision-making and surgical quality assessment.

## Data Availability

The datasets generated and/or analyzed during the current study are not publicly available due to patient privacy and confidentiality concerns, as well as the conditions stipulated by the ethical approval and data use agreement. However, de-identified data can be made available from the corresponding author upon reasonable request, subject to approval of a research proposal and a data sharing agreement. Requests to access the datasets should be directed to JH, hujiawei0714@163.com.
